# Giant-cell arteritis on photon-counting detector CT: A case report

**DOI:** 10.1016/j.radcr.2024.07.122

**Published:** 2024-08-10

**Authors:** Shunsuke Shibata, Misugi Urano, Nobuo Kitera, Haruka Noda, Shuntaro Isogai, Wenya Zhao, Toshihide Itoh, Tatsuya Kawai, Akio Hiwatashi

**Affiliations:** a Department of Radiology, Nagoya City University Graduate School of Medical Sciences, Nagoya, Japan; b Department of Radiology, Nagoya City University Hospital, Nagoya, Japan; c Division of Rheumatology, Department of Internal Medicine, Nagoya City University Hospital, Nagoya, Japan; d Department of CT-Research and Collaboration, Siemens Healthinieers, Tokyo, Japan

**Keywords:** Giant cell arteritis, Photon-counting detector CT, Cinematic rendering image, Superficial temporal artery, Maxillary artery

## Abstract

A 77-year-old woman presented to our hospital with a 2-week history of fever, headache, and induration along the bilateral superficial temporal arteries (STAs). The color Doppler ultrasonography of the STA showed a hypoechoic mural thickening surrounding a residual color flow. A contrast-enhanced photon-counting detector (PCD) CT demonstrated mural thickening and stenosis of the bilateral STAs. The patient underwent a biopsy of the right STA. Histopathological findings were consistent with giant cell arteritis (GCA). The patient's symptoms were temporarily relieved after initiation of steroid treatment, but jaw claudication occurred 2 months later. Contrast-enhanced CT showed improved vascular abnormalities of the STAs but new mural thickening and stenosis of the bilateral maxillary artery. Due to its higher resolution, image contrast, and lower noise, PCD-CT may have great potential in detecting, diagnosing, and monitoring GCA.

## Introduction

Giant cell arteritis (GCA) is the primary systemic large-vessel vasculitis affecting older patients [1,2]. Patients with GCA present with fever, headache, pain, and fatigue. GCA may induce arterial stenosis, occlusion, or aneurysm [2,3]. These conditions can lead to severe disease-related consequences, including stroke, blindness, and rupture of aortic aneurysm [4]. Therefore, early diagnosis and treatment of GCA are required in patients with suspected GCA.

Ultrasonography, CT, magnetic resonance imaging (MRI), or positron emission tomography/computed tomography (PET/CT) can be used for the assessment of vessel involvement in patients with suspected GCA [[5], [6], [7]]. Ultrasonography is recommended to investigate superficial arteries as the first imaging modality. CT is preferred in certain situations because of its lower acquisition times and shorter waiting times [7]. The role of CT is limited to detecting mural inflammation or luminal changes of extracranial arteries for mainly large vessels, such as the aorta and its first and second branches.

Photon counting detector CT (PCD-CT) produces images with high spatial resolution while reducing noise and improving image contrast. It may deliver more precise microvessel images than conventional energy-integrating detector CT (EID-CT) [8]. The present study describes a case of GCA illustrating mural thickening and stenosis of the superficial temporal arteries (STA) and maxillary artery (MA).

## Case

A 77-year-old woman visited our institution, presenting with fever, headache, and arthralgia, which had lasted 2 weeks. Physical examination revealed indurations along the bilateral STAs. No other specific findings, such as an inter-arm difference in blood pressure or myalgia, were shown.

**Fig. 1:**
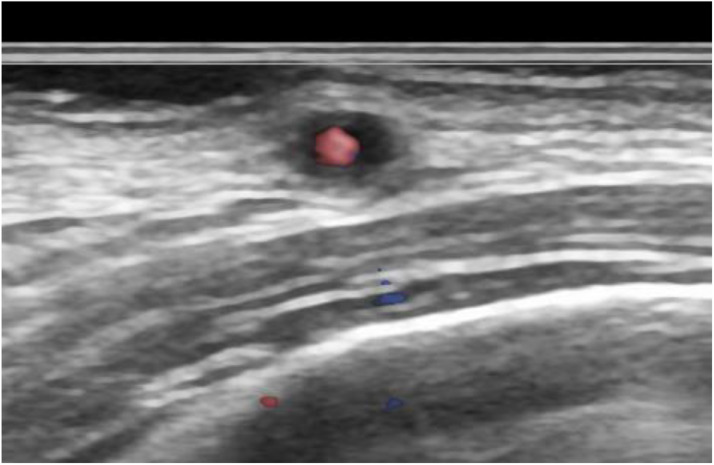
Transverse view of color Doppler ultrasonography depicting the superficial temporal artery. A “halo sign,” consisting of a uniformly hypoechoic thickened wall encircling residual color flow, is visible.

**Fig. 2:**
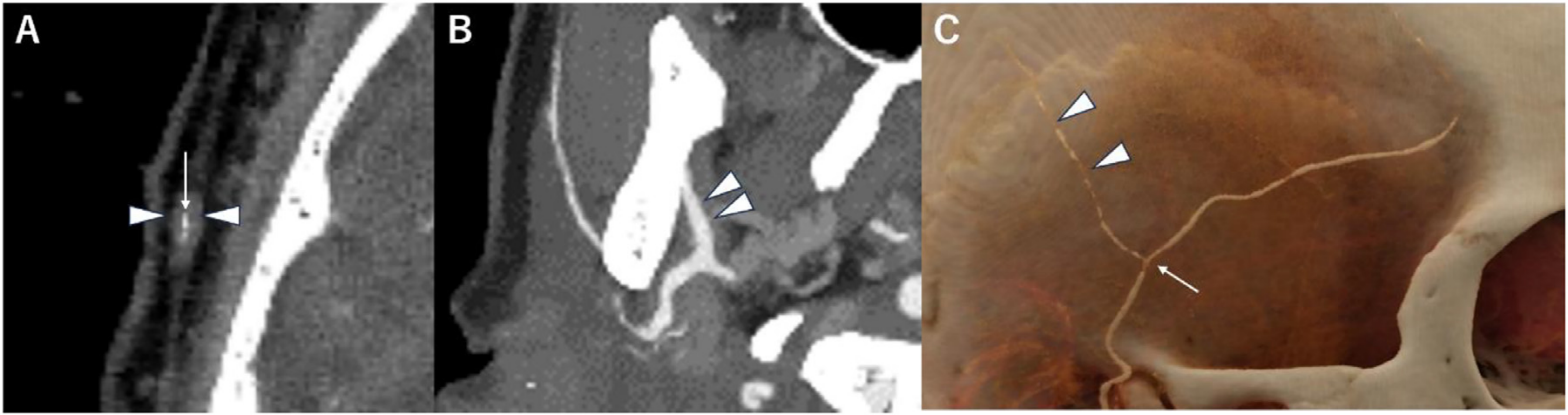
(A) CT angiography at the bifurcation of the frontal and parietal branches of the right superficial temporal artery shows a poorly enhancing thickened wall (arrowheads) with an enhancing luminal stenosis (arrow). (B) The thin-slab maximum intensity projection image at the maxillary artery shows no stenosis (arrowheads). (C) The cinematic rendering image depicts multiple stenoses of the parietal branch (arrowheads) and the bifurcation of the frontal and parietal superficial temporal artery branches(arrow).

**Fig. 3:**
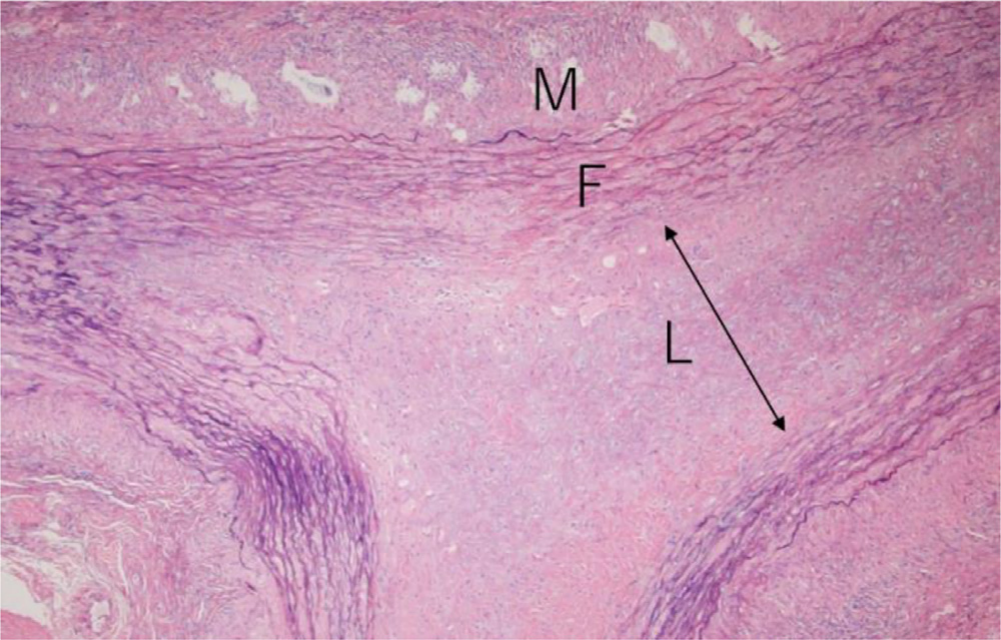
The histopathology of hematoxylin and eosin-stained sections of the right superficial temporal artery shows giant cell arteritis. It demonstrates infiltrating lymphocytes, neutrophils, plasma cells, multinucleated giant cells, and fibrosis (F) in the adventitial layer up to the muscular layer (M). The tissue organization forms a narrowed intravascular lumen (L).

The color Doppler ultrasonography of the STA showed a homogeneous*,* hypoechoic mural thickening deemed a 'halo sign' surrounding a residual color flow (Fig. 1). There was no abnormality in the axillary and subclavian arteries. A physician scheduled a contrast-enhanced CT examination due to suspicion of a diagnosis of GCA. CT was performed using a PCD-CT system (NAEOTOM Alpha, Siemens Healthineers, Forchheim, Germany) in the spiral acquisition mode with 0.2 mm, a spiral pitch of 0.85, tube voltage of 120 kV, and CT dose index volume of 18.7 (mGy). The scan range was from the head to the pelvis. After a noncontrast enhanced scan, intravenous access was secured by placing a 22-gauge intravenous line in the antecubital vein. A bolus administration of the contrast agent was performed by injecting 75 mL of 300-mgI/mL nonionic iodine contrast agent (Iopromide 300, Bayer, Nordrhein-Westfalen, Germany) at a rate of 2.9 mL/second using a power injector. The bolus-tracking technique was used to trigger the initiation of image acquisition, with region of interest (ROI) placement in the ascending aorta. The ROI threshold was set at 100 Hounsfield units. The delay times of the scans for the arterial and delayed phases were 4 and 100 seconds, respectively. Spectral CT scans were reconstructed with slice thicknesses of 0.2 and 5 mm for arterial and delayed phases, respectively, a Qr72 kernel and an iterative reconstruction strength of 3 regarding the arterial phase. CT data were sent to a dedicated workstation (syngo. via VB60A, Siemens Healthineers, Forchheim, Germany) to create thin-slab maximum intensity projection images with 5 mm thickness and cinematic rendering images of CT angiography (CTA) in the early phase. CTA showed mural thickening and stenosis at the right STA (Fig. 2A). However, no mural thickening and stenosis at MA at this time (Fig. 2B). The cinematic rendering images revealed stenotic lesions along the right STA in multiple segments (Fig. 2C). The patient underwent a biopsy from the right STA with a strong suspicion of GCA, based on the clinical and imaging findings. Histopathological findings revealed infiltrating lymphocytes, neutrophils, plasma cells, and multinucleated giant cells throughout the adventitial to the muscular layers, contributing to the narrowing of the intravascular lumen (Fig. 3). These findings were compatible with GCA.

**Fig. 4:**
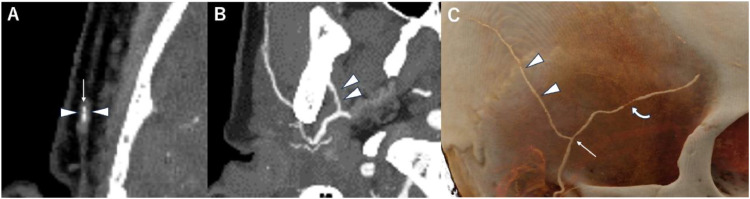
CT 2 months after initiation of steroid treatment. (A) CT angiography at the bifurcation of the frontal and parietal branches of the right superficial temporal artery shows a slight improvement of wall thickening (arrowheads) with residual luminal stenosis (arrow). (B) A thin-slab maximum intensity projection image at the maxillary artery demonstrates luminal stenosis (arrowheads). (C) The cinematic rendering image demonstrates an improvement in stenosis at the bifurcation (arrow) and the parietal branch of the right STA (arrowheads). Additionally, it reveals the emergence of stenosis at the frontal branch (curved arrow).

Two months after the initiation of treatment with steroids, the patient's symptoms were temporarily alleviated. However, a jaw claudication appeared with C-reactive protein level elevation 1 month after the alleviation. The follow-up PCD-CT showed improved vessel abnormalities in the bilateral STA and new stenoses of the bilateral MA (Fig. 4A, B). Cinematic rendering images revealed improved stenosis at the bifurcation and the parietal branch of the right STA, while the stenosis was worsened in the frontal branch (Fig. 4C). Thus, the patient was diagnosed with GCA relapse. The patient responded well to the additional treatment of methotrexate, resulting in improved symptoms.

## Discussion

GCA is a large vessel disease in elderly patients, affecting the aorta and its major branches, and the medium- and small-sized vessels [1,2]. Regarding extracranial vessels, GCA mainly affects external carotid artery branches, including STA, MA, occipital, and ophthalmic arteries. This case report represents the first instance to visualize GCA lesions at the STA and MA on PCD-CT for diagnosis and monitoring. The PCD-CT in the present case depicted a separation between the thickened vessel wall and the narrowed lumen in peripheral microvessels. Conway et al. [9] reported in a study using contrast enhanced EID-CT that one of the characteristic findings of GCA was a loss of marginal delineation of the vessel and an ill-defined perivascular hyperenhancing area. The differences in radiological findings between the previous report and our case might be due to higher spatial resolution, lower noise, and improved image contrast of PCD-CT than EID-CT [8]. In our case, mural thickening and luminal stenosis of the MA coincided with the onset of jaw claudication. This correlation between abnormal findings in the culprit vessels on PCD-CT and associated symptoms, such as the ophthalmic artery involvement and ocular symptoms, as well as the lingual artery involvement and tongue claudication, could prove beneficial in monitoring activity of GCA.

The European League Against Rheumatism (EULAR) recommends ultrasound as the primary imaging modality in patients with suspected GCA [7]. EULAR listed CT as the last in the list of recommended imaging modalities due to the lack of evidence that CT is better than MRI while exposing the patient to radiation. For long-term monitoring of structural damage, the choice of imaging modality, including MR angiography, CTA, and ultrasonography, depends on the vessels involved, the local setting, and the experts. Radiation exposure is considered a disadvantage, but PCD-CT reduces the radiation exposure [8]. Therefore, this is less of an issue than with conventional EID-CT. PCD-CT enables visualization of mural thickening and luminal changes of multiple arteries simultaneously in a shorter procedural time than MRI or PET/CT [7]. Our case suggests the clinical potential of high-resolution PCD-CT for detecting, diagnosing, and monitoring GCA.

## Conclusion

PCD-CT effectively visualized the STA and MA involved in GCA. Due to its higher resolution, image contrast, and lower noise, PCD-CT may have significant potential for detection, diagnosis, and monitoring of GCA.

## Patient consent

Informed consent was obtained for patient information to be published in this article.
